# Microbial Analyses of Ancient Ice Core Sections from Greenland and Antarctica

**DOI:** 10.3390/biology2010206

**Published:** 2013-01-25

**Authors:** Caitlin Knowlton, Ram Veerapaneni, Tom D’Elia, Scott O. Rogers

**Affiliations:** 1 Department of Biological Sciences, Bowling Green State University, Bowling Green, OH 43403, USA; E-Mail: cknowlt@bgsu.edu; 2 Department of Biological Sciences, Bowling Green State University, Firelands Campus, Huron, OH 44839, USA; E-Mail: ramv@bgsu.edu; 3 Biological Sciences, Indian River State College, 32021 Virginia Avenue, Fort Pierce, FL 34981, USA; E-Mail: tdelia@irsc.edu

**Keywords:** glacial ice, microbes, bacteria, fungi, Greenland, Antarctica, metagenomic, metatranscriptomic, cultures

## Abstract

Ice deposited in Greenland and Antarctica entraps viable and nonviable microbes, as well as biomolecules, that become temporal atmospheric records. Five sections (estimated to be 500, 10,500, 57,000, 105,000 and 157,000 years before present, ybp) from the GISP2D (Greenland) ice core, three sections (500, 30,000 and 70,000 ybp) from the Byrd ice core, and four sections from the Vostok 5G (Antarctica) ice core (10,500, 57,000, 105,000 and 105,000 ybp) were studied by scanning electron microscopy, cultivation and rRNA gene sequencing. Bacterial and fungal isolates were recovered from 10 of the 12 sections. The highest numbers of isolates were found in ice core sections that were deposited during times of low atmospheric CO_2_, low global temperatures and low levels of atmospheric dust. Two of the sections (GISP2D at 10,500 and 157,000 ybp) also were examined using metagenomic/metatranscriptomic methods. These results indicated that sequences from microbes common to arid and saline soils were deposited in the ice during a time of low temperature, low atmospheric CO_2_ and high dust levels. Members of Firmicutes and Cyanobacteria were the most prevalent bacteria, while *Rhodotorula* species were the most common eukaryotic representatives. Isolates of *Bacillus*, *Rhodotorula*, *Alternaria* and members of the Davidiellaceae were isolated from both Greenland and Antarctica sections of the same age, although the sequences differed between the two polar regions.

## 1. Introduction

Microorganisms have been identified and isolated from glacial ice samples from many different regions of the world. The global distribution of these microorganisms in snow and glacial ice is the result of wind and atmospheric circulation. Bacteria and fungi have been detected and isolated from many different frozen environments such as permafrost, cryopegs, sea ice, glacial ice, and accretion ice from subglacial lakes [1,2,3,4,5,6,7,8,9,10,11,12,13,14,15,16,17]. Bacteria and fungi have been isolated from both Arctic and Antarctic regions [4,5,7,8,9]. Most of the bacteria and fungi isolated from these permanently cold environments were psychrotolerant as opposed to psychrophilic, having optimal growth temperatures well above freezing [4,5]. Glacial ice has constant temperatures, making it ideal for long-term preservation of microorganisms and biomolecules [16,17,18,19]. Environmental ice acts as a protective matrix for microorganisms over extended periods of time and provides a record of microbial evolution and ancient biodiversity [8].

While the numbers of viable organisms and intact nucleic acid sequences decline with depth, they have been recovered from ice that is more than a million years old [4,5,14,16,17,18,20]. Study of the organisms isolated from environmental ice has yielded insights into microbial longevity. However, studies have not been performed to compare microorganisms belonging to similar timescales entrapped in glacial ice from geographically distant sites. The Arctic and the Antarctic are geographically distant regions, but also are distinct for other reasons. Much of Antarctica is a desert with little precipitation, and the continent is far from other large landmasses. In general, Greenland receives more precipitation, and is geographically less isolated, receiving winds from Europe, Asia and North America [21]. Study of microbes isolated from these two distinct locations, dating back to similar time periods may provide insights into microbial community composition through time and transportation of microbes in the atmosphere, as well as deposition and preservation of microbes and biomolecules in ice.

**Figure 1:**
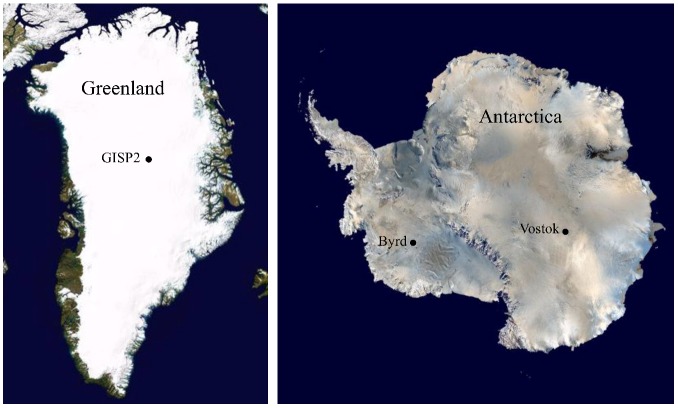
Locations of ice core sections used in this study. Sections from the Greenland Ice Sheet Project 2 (GISP2) (Greenland), Byrd (Antarctica) and Vostok 5G (Antarctica) ice cores were examined. See Experimental Section for more details about the core sections.

Sections from the GISP2D (Greenland Ice Sheet Project 2), Vostok 5G (Antarctica) and Byrd (Antarctica) ice cores were selected for comparison (Figure 1). Sections were chosen that were representative of times of high and low atmospheric carbon dioxide, dust and mean global temperature [22,23,24,25,26]. Scanning electron microscopy, cultivation, sequencing and phylogenetic analyses were used to assess the influences of atmospheric characteristics on microbe deposition and survival in ice. Metagenomic/metatranscriptomic analyses were used to determine and compare the microbial communities at times when carbon dioxide levels and temperatures were at minima (157,000 ybp) [22,23] and at a time when carbon dioxide levels and temperatures were rising nearly to modern levels (10,500 ybp) [22,23,24,25,26]. During the past 420,000 years, the concentration of atmospheric carbon dioxide has ranged from a low of approximately 200 ppm 150,000 years ago [22,23] to a high of >390 ppm currently [27], with another high peak of approximately 280 ppm occurring approximately 125,000 years ago [23]. Carbon dioxide levels vary directly with changes in global temperature. Low CO_2_ levels are normally associated with low temperatures, low precipitation and high dust levels, while high CO_2_ levels generally indicate high temperatures, high precipitation rates, and low dust levels, factors that may influence transportation of microbes and overall community composition. Here we report research that was designed to test whether the same species are concurrently deposited in ice at both poles, and whether changes in atmospheric carbon dioxide, dust and global temperature affect deposition of microbes in glacial ice.

## 2. Results and Discussion

### 2.1. Results

#### 2.1.1. Scanning Electron Microscopy (SEM)

**Figure 2:**
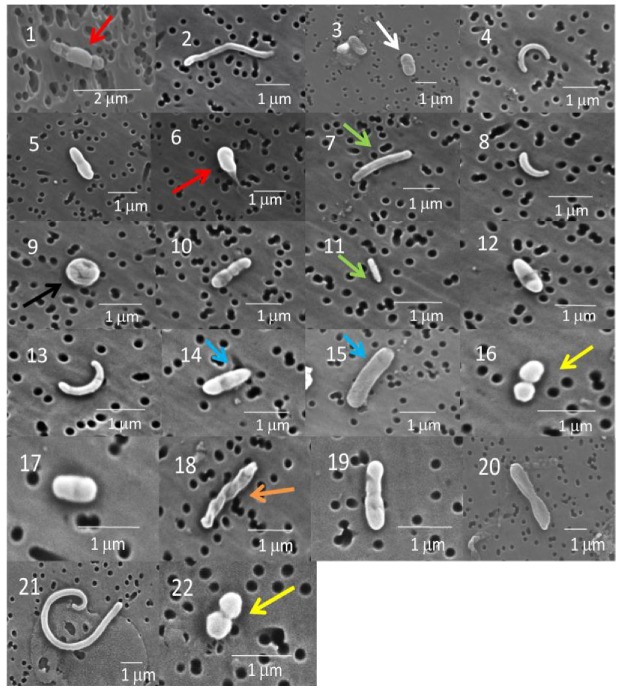
Scanning electron micrographs (SEM) of bacteria from the GISP2D ice core sections (panels 1–6, 1,601 m; 7–11, 2,501 m; 12–18, 2,777 m; 19–22, 3,014 m). Microbes in micrographs 1, 3, 5 and 6 are rod shaped bacteria. The microbes in micrographs 1 and 6 have a sheath-like cover over the exterior, similar to the microbes previously observed in Vostok accretion ice (red arrow; [4]). The organisms in micrograph 3 are similar to the rod shaped bacteria observed in previous GISP2D sediment ice studies (white arrow; [28]). Microbes in micrographs 7 and 11 are similar to long rod shaped bacteria observed in the Vostok ice cores (green arrow; [4]). Organisms in micrograph 14 and 15 are rod shaped bacteria, similar to those previously observed from Vostok ice cores (blue arrow; [4]). The organism in micrograph 16 has a diplococcoid form (yellow arrow) and has been observed in other studies of the Vostok and GISP2D ice cores and from several cold environments including the Siberian permafrost [4,14,17]. This microbe is similar to *Psychrobacter*, a diplococcoid bacterium commonly found in the cold environments. The microbe in micrograph 18 has an unusual spiral structure (orange arrow) similar to organisms isolated from the Vostok core [4]. These may be related to the order Spirochaetales, which have long helically coiled cells. The microbes in micrographs 8 and 13 resemble cells of *Caulobacter* spp. while the curving form in micrograph 21 cannot be identified.

**Figure 3:**
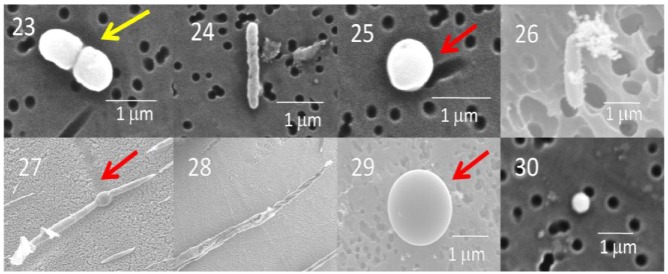
Scanning electron micrographs of bacteria from the Vostok 5G ice core section (micrographs 23–25, 316 m; 26–27, 900 m; 28–30, 1,529 m). No organisms were observed in the Vostok 5G 2,149 m ice core. Also, no cultures were obtained from this core section (Table 1). Diplococcoid organisms similar to the ones observed in the GISP2D cores were evident in micrograph 23 (yellow arrow). The microbe in micrograph 24 has a spiral structure and may be related to Spirochaetales. Spore-like structures were observed commonly (red arrow). A fungal hyphae-like structure was observed in the Vostok 5G 900 m ice core (panel 27). The spherical form in micrograph 29 appears to be a fungal coniduim. The microbes in Panels 25 and 30 are very small, similar to ultramicrobacteria found previously in the GISP2D ice core [28,29].

Most of the cells in the GISP2D and Vostok 5G core sections that were examined were rod-shaped bacteria (Figure 2 and Figure 3). A few were coccoid, including diplococcoid forms (Figure 2, panels 16 and 22; Figure 3, panel 23). Two were spiral shaped (Figure 2, panels 18 and 19). Only three appeared to be fungi (Figure 3, panels 27–29), more complex shapes and larger cells, some of which may be conidia.

#### 2.1.2. Revival and Molecular Identification of Glacial Isolates

**Figure 4:**
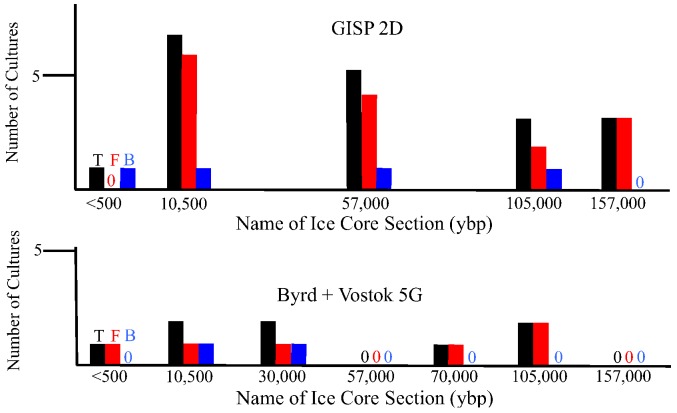
Number of cultures from GISP2D, Byrd and Vostok 5G ice core sections, based on age of ice. Black bars indicate total number of cultures, red bars indicate fungal cultures and blue bars indicate bacterial cultures.

**Figure 5:**
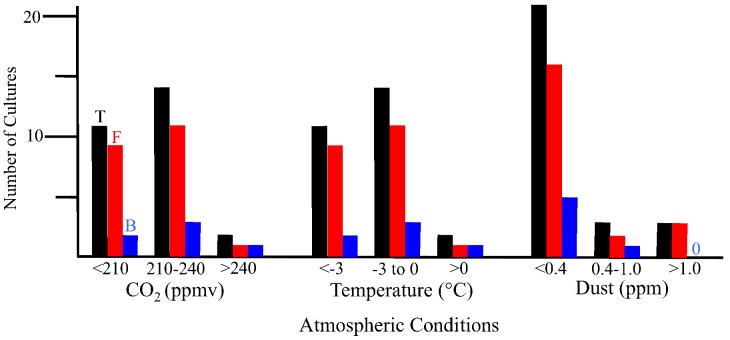
Cultures obtained from ice core sections based on atmospheric conditions at the time of deposition. Black bars indicate total number of cultures, red bars indicate fungal cultures and blue bars indicate bacterial cultures. Values for atmospheric measurements are from reference [23].

There are geographical and temporal differences in the microbial composition of ice core samples (Table 1). Of the 27 cultures recovered from the 10 ice core sections, 19 were from Greenland ice and only eight were from Antarctica ice. Also, in general, the number of viable microbes, as indicated by number of cultures, was inversely proportional to the age of the ice core (Figure 4). The only exceptions to this were for the youngest ice sections that were analyzed, which were less than 500 years old (from Byrd and GISP2D). In both cases, few cultures were obtained. The ice core sections that were 10,500 years old from the GISP2D (1,601 m) and Vostok 5G (316 m) cores yielded higher numbers of cultures (seven fungi and two bacteria; Table 1). The number of cultures decreased with increasing age of the ice core sections (Table 1, Figure 4). There was a correlation between the number of cultures obtained and the atmospheric conditions at the time of ice deposition (Figure 5). Higher numbers of fungi and bacteria were isolated when atmospheric CO_2_ levels were below 240 ppmv (parts per million per volume), temperatures were at least 3 °C lower than the present mean global temperature, and dust levels were <0.4 ppm (atmospheric measurements are from [23]).

**Table 1:** Summary of BLAST search results. Included are sequences from cultures and metagenomic/metatranscriptomic analyses.

Core	Depth (m)	Age (ybp)	Sequence number ^a^	Accession number	Closest BLASTn match ^b^	Phylum ^c^	Identity(%)
Byrd	99	<500	**CULT C-2**	KC146580	*Alternaria* sp.	As	99 ^d^
	1,593	30,000	**CULT C-8**	KC146581	*Alternaria alternata*	As	99 ^d^
			**CULT A-1**	KC146555	*Bacillus lichniformis*	Fi	99 ^d^
	2,131	70,000	**CULT D-7**	KC146582	*Alternaria alternata*	As	99 ^d^
GISP 2D	104	<500	**CULT B-1**	KC146556	*Bacillus thioparans*	Fi	100 ^e^
	1,601	10,500	**GI860**	KC206482	*Alternaria alternata*	As	100 ^d^
			c2581	KC155352	*Lactobacillus helveticus*	Fi	100 ^d^
			s3382	KC146557	*Microcoleus vaginatus*	Cy	99 ^e^
			c797	KC146560	*Microcoleus vaginatus*	Cy	99 ^e^
			c1832	KC146558	Uncultured bacterium	Cy	99 ^e^
			**GI859**	KC206491	*Fusarium culmorum*	As	98 ^d^
			s1867	KC155351	*Lactobacillus crispatus*	Fi	98 ^e^
			**GI862**	KC206492	*Penicillium corylophilum*	As	98 ^d^
			**GI867**	KC206485	*Rhodotorula mucilacinosa*	Ba	98 ^e^
			**GI861**	KC206488	*Cladosporium tennuissimum*	As	97 ^d^
			**GI866**	KC206494	*Rhodotorula mucilacinosa*	Ba	97 ^d^
			c2097	KC146554	Uncultured cyanobacterium	Cy	95 ^e^
			**GI865**	KC206477	*Caulobacter crescentus*	Ap	94 ^e^
			c4392	KC146571	*Escherichia coli*	Gp	93 ^e^
			s3934	KC146559	Uncultured bacterium	Ac	93 ^e^
			c3088	KC146584	*Halomonas neptunia*	Gp	92 ^e^
	2,501	57,000	**GI868**	KC206495	*Aspergillus restrictus*	As	99 ^d^
			**GI869**	KC206484	*Aureobasidium pullulans*	Ba	99 ^d^
			**GI871**	KC206489	*Aspergillus conicus*	As	98 ^d^
			**GI873**	KC206481	*Rhodotorula mucilacinosa*	Ba	98 ^d^
			**GI872**	KC206478	*Caulobacter crescentus*	Ap	86 ^e^
	2,777	105,000	**GI875**	KC206483	*Cryptococcus magnus*	Ba	99 ^d^
			**GI876**	KC206487	*Rhodotorula mucilacinosa*	Ba	99 ^d^
			**GI874**	KC209502	*Bacillus subtilis*	Fi	98 ^e^
	3,014	157,000	c2575	KC146576	*Lactobacillus helveticus*	Fi	100 ^f^
			c4301	KC146578	*Lactobacillus helveticus*	Fi	100 ^f^
			c1729	KC146569	*Micrococcus luteus*	Ac	100 ^f^
			**CULT E-5**	KC146583	*Cladosporium tenuissimum*	As	99 ^d^
			s3361	KC146561	*Microcoleus vaginatus*	Cy	99 ^e^
			c1654	KC146563	*Microcoleus vaginatus*	Cy	99 ^e^
			c552	KC146564	Uncultured bacterium	Cy	99 ^e^
			c1812	KC146565	Uncultured bacterium	Cy	99 ^e^
			c2330	KC146567	Uncultured bacterium	Fi	99 ^e^
			c785	KC146568	Uncultured bacterium	Cy	99 ^e^
			c3738	KC146577	*Lactobacillus helveticus*	Fi	98 ^f^
			c833	KC146573	*Lactobacillus helveticus*	Fi	98 ^e^
			c1018	KC146574	*Lactobacillus helveticus*	Fi	98 ^f^
			**GI855**	KC206493	*Penicillium chrysognum*	As	98 ^d^
			**GI858**	KC206480	*Rhodotorula mucilacinosa*	Ba	98 ^d^
			c1826	KC146566	Uncultured bacterium	Fi	98 ^e^
			c3135	KC146552	Uncultured cyanobacterium	Cy	98 ^e^
			c3856	KC146553	Uncultured cyanobacterium	Cy	98 ^e^
			s1137	KC146575	*Lactobacillus helveticus*	Fi	97 ^e^
			c2005	KC146570	*Micrococcus luteus*	Ac	97 ^e^
			c67	KC146562	*Microcoleus vaginatus*	Cy	97 ^e^
			c3509	KC146585	*Brevudimonas diminuta*	Ap	95 ^f^
			c3833	KC146587	Uncultured bacterium	Bp	93 ^e^
			c2683	KC146579	*Pseudomonas stutzeri*	Gp	91 ^f^
			c4709	KC146572	*Bordetella petrii*	Bp	89 ^f^
			s3724	KC146586	*Bordetella pertussis*	Bp	88 ^f^
			c2094	KC146588	*Geobacillus* sp.	Fi	84 ^g^
Vostok 5G	316	10,500	**GI878**		*Bacillus amyloliquifaciens*	Fi	100 ^e^
			**GI877**		*Davidiella tassiana*	As	99 ^d^
	900	57,000	None		----------	--	----------
	1,529	105,000	**GI879**		*Alternaria tenuissimum*	As	100 ^d^
			**GI880**		*Rhodotorula mucilacinosa*	Ba	99 ^d^
	2,149	157,000	None		----------	--	----------

^a^ Cultures are indicated in bold font. Metagenomic/metatranscriptomic sequences are in standard font. ^b^ Fungi are in red font. ^c^ Phyla: Ac—Actinobacteria; Ap—Alphaproteobacteria; As—Ascomycota; Ba—Basidiomycota; Bp—Betaproteobacteria; Cy—Cyanobacteria; Fi—Firmicutes; Gp—Gammaproteobacteria. ^d^ Ribosomal RNA internal transcribed spacer (ITS1 and ITS2). ^e^ Ribosomal RNA small subunit gene. ^f^ Ribosomal RNA large subunit gene. ^g^ Glycerol kinase gene.

#### 2.1.3. Metagenomic/metatranscriptomic Analyses

A total of 107,700 high quality 454 sequence reads were obtained from the sequencing facility. After categorization by the MID primers, there were 25,748 high quality reads associated with the MID4 (GISP2D, 1,601 m) primer, 29,506 associated with the MID5 (GISP2D, 3,014 m) primer. 18,015 high quality reads associated with the MID6 (control 1) primer, and 34,063 high quality reads associated with the MID10 (control 2) primer. After assembly, a total of 15,891 contigs resulted. Sequences that were in common with the control samples were removed, leaving 33 sequences that were unique to the ice for analysis (Table 1). Nine unique Basic Local Alignment Search Tool (BLAST) hits were present in the 10,500 ybp GISP2D core section and 24 unique hits were present in the 157,000 ybp core section. All of the metagenomic/metatranscriptomic sequences were from bacteria (Table 1). There were five species common to both samples. All others were unique to one sample or the other. In the 10,500 ybp sample, all nine sequences were unique, while in the 157,000 ybp sample, 15 species were indicated among the 24 sequences.

In general, the taxa in the cores were similar to organisms found in polar ice (both Arctic and Antarctic), soil, dust, water, hot springs, marine environments, deep-sea thermal vents and associated with animals. A number of isolates and sequences recovered from the ice in this study were those that have yet to be scientifically described. In the National Center for Biotechnology Information (NCBI) database, they are termed “environmental taxa” or “unidentified” or “uncultured.” In the 10,500 ybp ice, five of the sequences were closest to unidentified environmental taxa that were similar to *Microcoleus* and uncultured bacteria. Also, the 10,500 ybp GISP2D ice sample contained one sequence closest to a sequence of a *Halomonas* isolate that lives in marine environments, including deep-sea thermal vents. In the 157,000 ybp ice section sample, fifteen sequences were closest to those from unidentified environmental species. They were closest to members of *Geobacillus*, *Micrococcus*, *Microcoleus* and *Pseudomonas*. Five were associated with soil, three specifically with arid land soils, and two sequences were closest to *Micrococcus*, known to be saprotrophic.

#### 2.1.4. Phylogenetics

**Figure 6:**
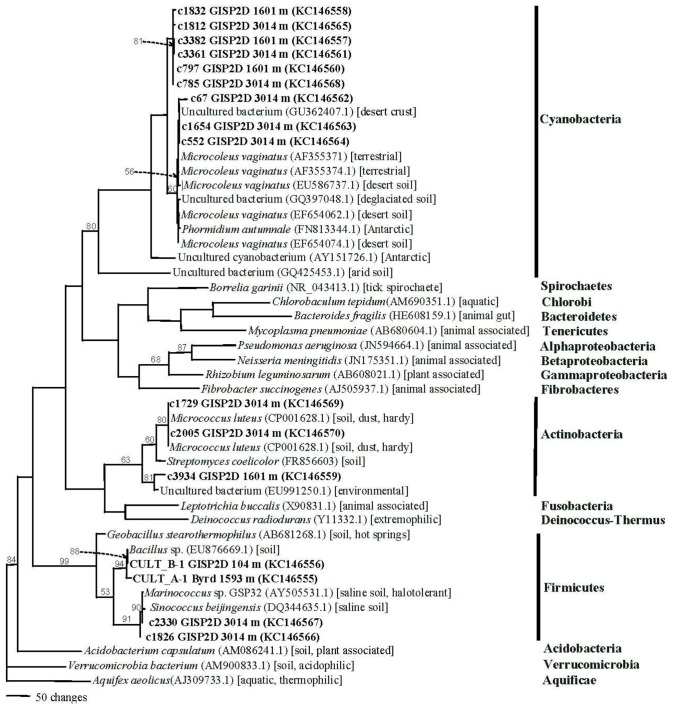
Phylogram of bacterial small subunit ribosomal RNA (SSU rRNA) sequences (maximum parsimony using phylogenetic analysis using parsimony (PAUP) [30]) from the GISP2D and Byrd ice cores. Sequences determined in this study are indicated in bold font. Sequences that start with CULT are isolates. All other sequences in bold were from the metagenomic/metatranscriptomic analysis. Ice core location and depth (meters below the glacial surface) are indicated. Phyla are on the right. The closest National Center for Biotechnology Information (NCBI) sequences to the queried sequences were selected from BLAST search results for use in the phylogenetic analysis. NCBI accession numbers (in parentheses) and sources of isolates (square brackets) are provided. Bootstrap values (1,000 replications) are given for branches with support greater than 50%.

**Figure 7:**
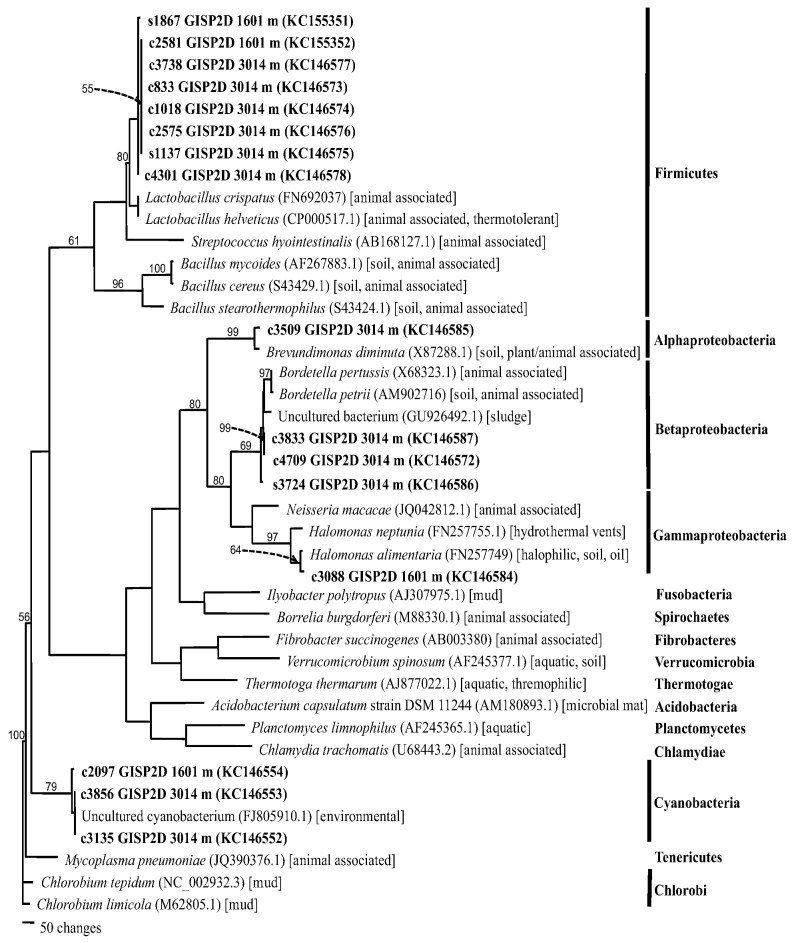
Phylogram of bacterial large subunit rRNA (LSU rRNA) sequences (maximum parsimony using PAUP [30]) from the GISP2D ice core. Sequences determined in this study are indicated in bold font. Ice core location and depth (meters below the glacial surface) are indicated for each. Phyla are on the right. The closest NCBI sequences to the queried sequences were selected from BLAST search results for use in the phylogenetic analysis. NCBI accession numbers (in parentheses) and sources of isolates (square brackets) are provided. Bootstrap values (1,000 replications) are given for branches with support greater than 50%.

For bacteria, phylogenetic trees were produced using SSU rRNA (small subunit ribosomal RNA; Figure 6) and LSU rRNA (large subunit rRNA; Figure 7) sequences. For fungi, the rRNA internal transcribed spacers (ITS1 and ITS2) were used, due to their increased precision for species determination for Eukarya. Phylogenetics was used to confirm the BLAST search results, as well as to refine species identities. The bacteria grouped into four clades, corresponding to phyla: Actinobacteria, Cyanobacteria, Firmicutes, and Proteobacteria (including Alphaproteobacteria, Betaproteobacteria and Gammaproteobacteria), the majority being in either the Cyanobacteria or the Firmicutes. Three sequences from the 157,000 year old ice core were closest to sequences from *Microcoleus vaginatus* and *Phormidium autumnale* that were isolated from soils, desert soils or deglaciated soil (including one from Antarctica). The other six sequences formed a separate Cyanobacteria clade, which was within the larger clade that included sequences from other Antarctic and arid soil taxa. The Actinobacteria clade included three sequences, all closely allied with sequences from environmental samples from soil or dust. Two were closest to sequences from *Micrococcus luteus*. Four sequences grouped within the Firmicutes clade. Two were from cultures whose sequences were within the genus *Bacillus*. In previous studies of Greenland ice cores, several *Bacillus* spp. were isolated [3]. Two metagenomic/metatranscriptomic sequences from the 157,000 year old ice core section most closely matched sequences from *Marinococcus* sp. and *Sinococcus beijingensis*, both of which had been isolated from saline soils. This is consistent with the low temperature and high atmospheric dust concentration at that time [23].

Fewer sequences from Cyanobacteria were found in the LSU rRNA gene data set. However, they all were within a clade that included an uncultured cyanobacterial sequence from an unidentified environmental sample. Firmicute sequences predominated in the LSU rRNA data set. All were most closely allied with sequences from *Lactobacillus crispatus* and *L. helveticus*. Both are associated with animals, but similar taxa can be found in soils. Similarly, one Alphaproteobacterium and three Betaproteobacteria were most closely related to sequences from bacteria associated with animals, but which can also be found in soil and sludge. One sequence most closely matched sequences from Gammaproteobacteria. It was closest to members of the halophilic and thermotolerant genus *Halomonas*. Species of *Halomonas* and *Lactobacillus* are thermotolerant. Coincidentally, dark granular material was found in the 10,500-year-old ice core section that resembles volcanic ash (data not shown).

**Figure 8:**
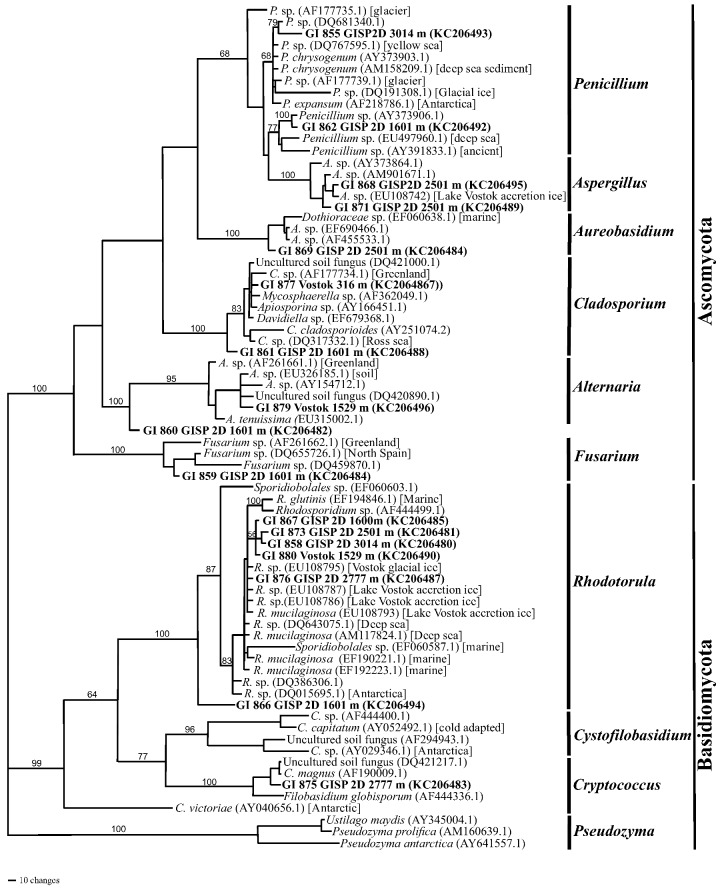
Phylogram of fungal rRNA internal transcribed spacer (ITS) sequences (with maximum parsimony on PAUP, [30]) from the GISP2D and Vostok 5G ice cores. Sequences that start with GI (in bold font) are the isolates from the ice sections. Ice core location and depth (meters below the glacial surface) are also given. Genera and phyla designations are on the right. Organisms belonging to the genera *Rhodotorula*, *Alternaria* and *Cladosporium* have previously been isolated from the GISP2D and the Vostok 5G ice cores. The closest NCBI sequences to the queried sequences were selected from BLAST search results for use in the phylogenetic analysis. NCBI accession numbers (in parentheses) and sources of isolates (square brackets) are provided. Bootstrap values (1,000 replications) are given for branches with support greater than 50%.

All of the fungi that were isolated and sequenced (internal transcribed spacer (ITS) regions) were either in the Basidiomycota or the Ascomycota. *Rhodotorula* was the most common genus (Figure 8). Several isolates (GI858, GI867, GI873 and GI876) were recovered from sections of the GISP2D ice core that were 10,500, 57,000 and 157,000 years old, as well as from a 105,000-year-old Vosok 5G ice core section (GI880). Most grouped closest to *R. mucilaginosa*, *R. glutius* and related isolates that had previously been recovered from marine, deep-sea or Lake Vostok ice samples. However, isolate GI866 was distant from all other isolates, as well as from sequences on NCBI. While it appears that it could be within the Sporidiobolales, it might be outside of the genus *Rhodotorula*.

Isolates of *Penicillium* and *Aspergillus* also were common. The ITS sequence from isolate GI855 (from GISP2D, 3,014 m) was closest to *P. chrysogenum* isolates from marine and deep-sea sediments. Another isolate (GI862, from GISP2D, 1,601 m) also was close to a deep-sea isolate. Two isolates (GI868 and GI871, both from Vostok 5G, 2,501 m) were closest to an isolate of *Aspergillus* from Lake Vostok accretion ice. All of these isolates, as well as the isolates described in the NCBI database originated from cold high-pressure environments. This is likely a common trait for organisms that are able to survive in deep ice. The sequence of isolate GI869 (from Vostok 5G, 2,501 m) is closest to marine isolate within the Dothioraceae, which includes the genus *Aureobasidium*. However, the sequence differs from all other such sequences in this family on the NCBI database, as indicted by the branch length (Figure 8). Sequences from two isolates (GI861, from GISP2D, and GI877, from Vosok 5G; both at 10,500 ybp) are within the *Cladosporium*/*Davidiella* complex. They were closest to marine and Greenland ice taxa [8].

One isolate of *Alternaria* was recovered from the 105,000-year-old section from Vostok 5G. The sequence was closest to *A. tenuissima*, as well as to isolates from Greenland and from soil. One isolate (GI859) was closest to sequences from *Fusarium* isolates recovered from Greenland and northern Spain. Isolate GI860 (from GISP2D, 1,601 m) could not be placed within a specific taxon. It appears to be approximately midway between *Alternaria* and *Fusarium* (Figure 8). One additional basidiomycete was isolate GI875 (from GIDP2D, 2,777 m), whose sequence is closest to *Cryptococcus magnus*, a species isolated from soil that is also an opportunistic pathogen of humans and other mammals.

### 2.2. Discussion

#### 2.2.1. Atmospheric Conditions and Microbial Deposition

Microbial cells are transported by wind, clouds and precipitation [12,31]. Some become entrapped in glaciers, becoming records of the atmosphere at specific points in time. In this study, two major questions are addressed: (1) Are the same species of microbes concurrently deposited in ice in the Arctic and Antarctic?, and (2) Do atmospheric conditions affect the deposition of microbes in polar ice? The answer to Question 1 is, no. The sequences found in the Arctic differed from those found during the same time periods in the Antarctic, although very similar sequences were sometimes found in ice from both polar regions (Table 1, Figure 6, Figure 7, Figure 8). The answer to Question 2 is, yes. The highest numbers of isolates were from ice core sections that contained ice deposited during times of moderate to low atmospheric CO_2_ (<240 ppmv), low temperature (<−3 °C below current global mean) and low dust (<0.4 ppm). These correlations to the cultivation results may indicate increased preservation of the microbes during times of colder global mean temperatures. The metagenomic/metatranscriptomic data indicated that sequences from microbes common to arid and saline soils were deposited in the ice during a time of low temperature, low atmospheric CO_2_ and high dust levels. The presence of nucleic acids does not indicate whether viable cells are present, and therefore, some of the organisms represented in these samples may have been nonviable. The metagenomic/metatranscriptomic data might be more indicative of the nucleic acids that are in the highest concentrations in the ice, rather than an indication of intact microbes.

The lower number of microbes in the Vostok sections could partly be due to the high elevation of the site and the fact that the Vostok site lies within a cold desert that receives much less snowfall compared to Greenland. Conversely, the higher number of microbes isolated from the Greenland ice cores may be related to a much warmer climate in the recent history compared to the Antarctic cores. During the Eemian interglacial (130,000 to 114,000 ybp) the northern hemisphere had warm temperatures comparable or higher to the temperatures of the Holocene period [32,33]. During this period, part of Greenland (southernmost part of Greenland) was covered with forests including a diverse array of conifers [34]. The warmer temperatures along with the higher precipitation and the geographically closer location to forests may be responsible for the higher number of organisms isolated from the Greenland ice cores. Distances to the nearest land masses with temperate climates likely contributed to the differences observed. The GISP2D site lies relatively close to North America, Europe and Asia, and winds often intersect the GISP2D site from these regions. On the other hand, most of the winds that reach the Vostok and Byrd sites originate in Antarctica, and the closest land mass is the narrowest part of South America. Therefore, the deposition of organisms from land masses onto Antarctica glaciers would be expected to be much less than onto Greenland glaciers. The numbers of cosmopolitan species isolated in Arctic and Antarctic regions along with spore trap data add credence to the point [35].

#### 2.2.2. Isolated Fungi and Bacteria

All of the bacterial and fungal isolates grew at 22 °C. These isolates do not fit the profile of psychrophiles, whose optimal growth temperature is below 15 °C and do not grow at temperatures above 20 °C. The isolates can be classified as psychrotolerant, as they can survive and grow at low temperatures while having the optimal and maximum growth temperatures above 20 °C. Earlier studies indicated that most of the polar ice habitats are dominated by psychrotolerant organisms [4,5,31,36]. Psychrophiles thrive and flourish in continuously cold environments like the sea ice where long periods of sustained cold temperatures act as the major selective pressure. However, in the polar regions, sharp and rapid changes in seasonal temperatures act as the main selective pressure. Significant environmental changes may kill many true psychrophiles, while generalists such as psychrotolerant species have better chances to survive. The organisms in these environments should be able to survive freeze thaw cycles that occur during the seasonal changes. Sharp changes in the surface temperatures have been observed in the Antarctic soils and have been recorded to go as high as 30 °C [37]. If fungi and bacteria from these surrounding environments are deposited in the glacial ice, we would expect to find psychrotolerant species among our isolates. It is important to note that the highest number of cultures were from ice core sections that were deposited during times of cold global temperature (e.g., 30,000, 70,000 and 157,000 ybp; Table 1, Figure 5). This might be caused by increased preservation of the microbes during transport and deposition due to the cold temperatures in the atmosphere.

Species from three genera were isolated from both Greenland and Antarctic ice: *Alternaria*, *Bacillus* and *Rhodotorula*. However, in all cases, the species, as indicated by the rRNA sequences, differed from one pole to the other (Table 1, Figure 6, Figure 7, Figure 8). The taxon that was most often isolated was the genus *Rhodotorula* (Table 1, Figure 8), as was the case in our previous studies of Greenland and Antarctic ice core sections [5,12]. Members of this genus are hardy and adaptable, and have been isolated frequently from polar regions [12]. All of the *Rhodotorula* isolates were pigmented (shades of yellow, orange, pink and red), which might be important to survival in cold environments, and might explain their abundance in the cores analyzed [38]. *Rhodotorula* spp. remain viable after many freeze thaw cycles, which is another explanation for the abundance in the ice cores analyzed [12].

Isolates from the Greenland core sections included sequences closely related to *Aspergillus* and *Penicillium*. Isolate KC206495 (99% similar to *Aspergillus restrictu*s) and KC206489 (99% similarity to *Aspergillus conicus*) were isolated from the 57,000-year-old ice core. Isolate KC206492 (98% similarity with *Penicillium corylophilum*) was isolated from the 10,500-year-old ice core and KC206493 (98% sequence similarity with *Penicillium chrysogenum*) was isolated from a 157,000-year-old ice core. Several species of *Penicillium* are tolerant to cold conditions and are known to survive for long periods of time [39]. Four of the isolates from Greenland ice cores were closely related to the species of *Cladosporium*, *Cryptococcus* and *Aureobasidium*. All have been frequently isolated from cold environments, such as Antarctic moss, which is one of the richest microhabitats for fungi [40]. *Cladosporium* spores have been found in high numbers in the air, especially in the temperate and tropical environments; the total fungal spore load in the Arctic region air is only about 5% [38]. Spore counts in the Antarctic atmosphere varied with the seasons, and highest spore counts were observed during the summer months, attributed to winds from northern regions [38]. One of the isolates from the Vostok ice core, one from GISP2D and three from the Byrd ice core were closely related to *Alternaria* sp. isolated from cryopegs in Siberia [15]. All of the cultured fungal sequences from this study had a 97% similarity or higher to NCBI sequences.

Several bacterial species isolated from the ice cores had high sequence similarities to the genera *Bacillus* and *Caulobacter*. Organisms belonging to these genera are tolerant to low temperatures and have been isolated from glacial and accretion ice samples from Lake Vostok [4]. Castello *et al*. [3] performed an extensive study of Greenland glacial ice (GISP2D and Dye 3) to report isolation of *Bacillus* through culturing meltwater. In a very similar method to theirs, we obtained our sequences related to *Bacillus*. All of the organisms isolated from the Arctic and Antarctic ice cores were closely related to species that are known to survive and thrive in cold environments and have been isolated from similar environments, indicating that the isolates probably are from the glacial ice and not contaminants.

#### 2.2.3. Metagenomic/Metatranscriptomic Analysis

While we saw a decrease in the number of viable microbes cultured from older ice (Figure 4), the metagenomic/metatranscriptomic results suggest that more nucleic acid (probably carried within living and dead microbes) deposition in glaciers occurred during periods of low CO_2_, lowered temperatures and high dust (e.g., 157,000 ybp). The cultivation results indicated that more viable microbes were present in ice that had been deposited in the glacier during periods of moderate to low CO_2_, moderate to low temperatures and low dust (e.g., 500, 10,500 and 105,000 ybp). Together, the results indicate that cold temperatures are needed to retain cell viability, and to lessen degradation of nucleic acids. While decreases in temperature and CO_2_ were positively correlated with the recovery of viable microbes, increases in the amount of dust were negatively correlated. There have been reports of a variety of organisms present in Greenland ice (e.g., Ma *et al*. [8,9]). It has been shown that more long-range transport and deposition of microbes occurs during times of lower temperature, but it has been assumed that dust particles are responsible for the increases in transport. The results presented here argue against this assumption. Rather, temperature might be more important than the presence of dust particles. Microorganisms can be deposited through precipitation. Reports of Actinobacteria, Firmicutes, Proteobacteria (primarily Alphaproteobacteria, Betaproteobacteria and Gammaproteobacteria) and Bacteroidetes, and genera such as, *Pseudomonas*, *Sphingomonas*, *Staphylococcus*, *Streptomyces* and *Arthrobacter* were found in tropospheric clouds [31]. These phyla are often found in cold environments, freshwater, marine water, soil or vegetation. Also, fog water bacteria consisted of *Pseudomonas*, *Bacillus*, *Actinetobacter* and several fungi [31]. It may be that most of the organisms isolated in this study were preserved and deposited in snow, not dust.

Another important component to long-term survival is the ability to survive cold and desiccation. An example of a microbe found in arid conditions that was found in the ice cores was *Microcoleus vaginatus* (99% identity). This bacterium is found most often in biocrusts of arid land [41,42,43]. The phylogenetic analyses based on the SSU rRNA gene sequences (Figure 6) indicated that the majority of microbes that were in the 157,000 year old ice originated from soils, especially those from deserts and saline soils, and that they were hardy and desiccation resistant. This is consistent with conditions at the time, which were dry and cold [23]. Cyanobacteria and Firmicutes were present in the Greenland (GISP2D) ice core sections (Figure 5 and Figure 6), including many that are similar to species from soil, arid soil and polar regions. Microorganisms in both hot and cold arid environments have mechanisms for adaptation to extremes in climate (temperature, salt levels and lack available water), such as accumulation of organic compounds that aid in resistance to desiccation and freezing [41]. Cyanobacteria have been reported to enrich the soil stability due to the protection and adaptability of the polysaccharide sheath that surrounds the filaments [42]. *Microcoleus vaginatus* is capable of synthesizing and degrading trehalose [41] from maltose and is highly motile [43]. Trehalose is a cryoprotectant that can help this organism survive in the ice [44,45]. Firmicutes surround themselves with sheaths that protect against desiccation, osmotic shock and temperature changes.

The results presented here confirm that an overwhelming majority of sequences in ancient ice are derived from bacteria [1,4,5,46], while the greatest number of isolates were fungi. This might be due to the relative numbers of total microbes to the total viable microbes in each core section, although rigorous testing of this supposition will have to be performed in subsequent research. There was a diverse assemblage of bacterial phyla in the two ice samples analyzed by metagenomics/metatranscriptomics. In general, it is consistent with previous research, which indicates that Proteobacteria and Actinobacteria are commonly present in glacial ice [47,48]. Castello *et al*. [3] reported finding a sequence related to *Rhodococcus erythreus*, similar to our two sequences closely related to *Micrococcus luteus*, which are also in the Actinobacteria. In one study by Christner *et al*. [49], molecular analysis of glacial ice from a variety of global locations indicated approximately 50% of organisms to be Gram-positive, spore forming organisms. This could be due to the thick layer of peptidoglycan that provides strength and protection of the cell. Many of the microbes found in this study also form spores. Studies have shown Actinobacteria, Firmicutes, Proteobacteria and Fungi to be in the highest proportions in ice cores [48]. In our analyses, the same taxa were also found, with the addition of Cyanobacteria. The number of members of the Cyanobacteria was lower in the 10,500 ybp core section than in the 157,000 ybp section. The reason for this remains unclear.

#### 2.2.4. Global Mixing, Gene Flow and Genome Recycling

Organisms were isolated from 10 of the 12 ice cores used. However, there were differences among the ice core sections. Ice cores from the Arctic region consistently contained more viable organisms than those from Antarctica. Furthermore, species differed in the Arctic and Antarctic, suggesting that atmospheric transport, gene flow and genome recycling [50] between the poles occur infrequently. It appears that the two polar regions entrap microbes only from geographically local regions. Therefore, it is likely that latitudinal global mixing, leading to the global distribution of single genotypes, is inefficient over the long distance between the polar regions. Additionally, there appears to be a temporal component to preservation and deposition in polar ice. Far more isolates and sequences were found in ice core sections that had been deposited during periods of global cooling, when dust levels also were low. Thus, local and global conditions appear to be influential in the patterns of preservation of microbes and nucleic acids in Arctic and Antarctic ice.

## 3. Experimental Section

### 3.1. Ice Core Selection and Samples

**Figure 9:**
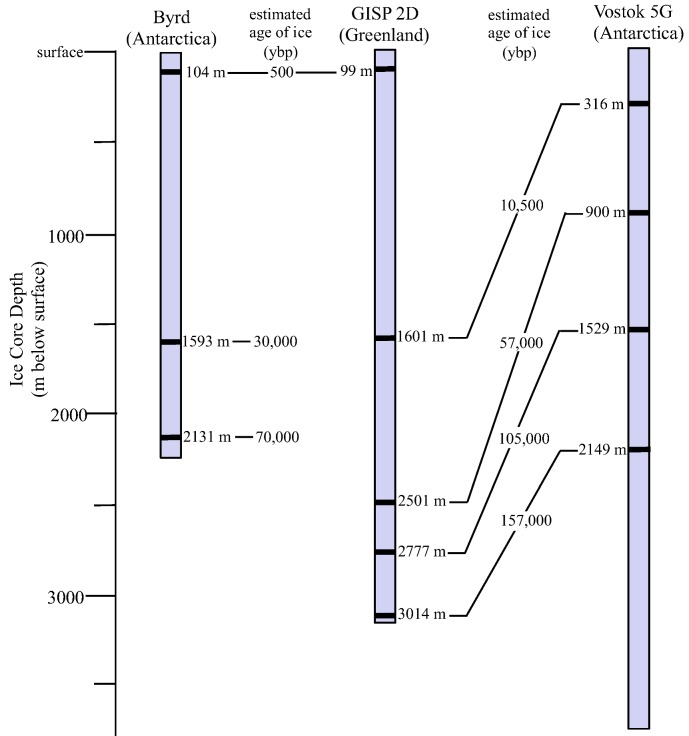
Depths and estimated ages of ice core sections used in this study. Atmospheric CO_2_ levels (all in ppmv): 500 ybp = 250; 10,500 and 105,000 ybp = 230; 70,000 ybp = 210; 30,000 = 200; 57,000 and 157,000 = 180. Global temperature: (relative to present temperature, in °C): 500 ybp = +2; 10,500 and 105,000 = −3; 57,000 = −4; 30,000, 70,000 and 157,000 = −6. Atmospheric dust (ppm): 500 = <0.1; 105,000 = 0.1; 10,500 and 57,000 = 0.2; 70,000 = 0.5; 30,000 = 0.7; 157,000 = 1.2. Values for atmospheric measurements are from reference [23]. All values are from reference [23].

Three ice core sections (104 m, 1,593 m, and 2,131 m; approximately 500, 30,000, and 70,000 ybp, respectively; [51]) from Byrd Station (80°1' S, 119°31' W, elevation 1,553 m), Antarctica; five core sections (99 m, 1,601 m, 2,501 m, 2,777 m and 3,014 m; approximately 500, 10,500, 57,000, 105,000 and 157,000 ybp, respectively [23]) from GISP 2D (Greenland Ice Sheet Project, core 2D; 72°36' N, 38°30' W, elevation 3,203 m); and four sections (316 m, 900 m, 1,529 m and 2,149 m; approximately 10,500, 57,000, 105,000 and 157,000 ybp, respectively [22,24,26]) from the Vostok 5G ice core (Vostok Station, Antarctica, 78°52' S, 106°50' W, elevation 3,488 m) were selected (Figure 1 and Figure 9). The ice cores sections were chosen based on atmospheric CO_2_, dust, and global temperature at the time of ice deposition [23].

### 3.2. Surface Sterilization and Melting

The outer surfaces of the ice core sections were decontaminated before melting. Clorox (a 5.25% sodium hypochlorite solution) was used as the decontaminating agent. The decontamination protocol developed by Rogers *et al*. [52] was used for this procedure.

### 3.3. Scanning Electron Microscopy

An aliquot (meltwater from ice cores) of 5 mL was filtered through a 0.2 μm polycarbonate filter using a sterile syringe. The filter was transferred to a Petri dish with 2.5% glutaraldehyde in 0.1 M phosphate buffer (pH 7.2). The filter was fixed in this solution for 1 h, followed by three 10 min rinses in 0.1 M phosphate buffer (pH 7.2). The filter was dehydrated with 40%, 60%, 80% and 95% ethanol solutions, sequentially for 10 min each and finally with 100% ethanol three times (10 min each). After dehydration, the filter was dried using a Samdri 780A critical point dryer. Then, the filter was cut into four pieces to be mounted into a coating unit. A Polaron E500 SEM coating unit was used to sputter coat the filters with a 5 nm thick gold-palladium coat. The filters then were observed in a scanning electron microscope (Hitachi S-2700, SEM). Control filters were prepared for SEM using sterilized water, taken through the fixation and dehydration procedure and observed.

### 3.4. Culturing

The meltwater (200 μL) from each shell of the ice cores was spread onto several types of solid media in duplicate, and incubated at 8 °C to test for the presence of viable microorganisms. The following media were used: malt extract agar [1.28% maltose, 0.27% dextrin, 0.24% glycerol, 0.08% peptone, 1.5% agar (pH 4.7)], potato dextrose agar [0.4% potato starch, 2% dextrose, 1.5% agar (pH 5.6)], rose bengal agar [0.5% soytone, 1% dextrose, 0.1% monopotassium phosphate, 0.005% rose bengal, 1.5% agar (pH 7.2)], nutrient agar [0.3% beef extract, 0.5% peptone, 1.5% agar (pH 6.8)], oatmeal agar [6% oatmeal, 1.25% agar (pH 6.0)], Sabouraud dextrose agar [1% enzymatic digest of casein, 2% dextrose, 2% agar (pH 7.0)], yeast extract agar [3% yeast extract, 3% malt extract, 0.5% peptone, 1% dextrose, 2% agar (pH 6.2)], acidic yeast extract agar [3% yeast extract, 3% malt extract, 0.5% peptone, 1% dextrose, 2% agar (pH 4.5)], meat-liver agar [2% meat liver base, 0.075% D(+)-glucose, 0.075% starch, 0.12% sodium sulfite, 0.05% ammonium ferric citrate, 1.1% agar (pH 7.6)], blood agar [1.5% pancreatic digest of casein, 0.5% papaic digest of soybean meal, 0.5% sodium chloride, 5% sheep’s blood, 1.5% agar (pH 7.3)], R2A [0.05% yeast extract, 0.05% proteose peptone No. 3, 0.05% casamino acids, 0.05% dextrose, 0.05% soluble starch, 0.03% sodium pyruvate, 0.03% dipotassium phosphate, 0.005% magnesium sulfate, 1.5% agar (pH 7.2)], Luria-Bertani agar [1% tryptone, 0.5% yeast extract, 0.5% sodium chloride, 1.5% agar (pH 7)], water agar [2% agar]. They were incubated for 2 weeks, monitored for any growth and transferred to 15 °C for 2 weeks. The plates then were incubated at 22 °C and monitored periodically for microbial growth. The cultures obtained were recorded and subcultured for future use. Throughout the decontamination and culturing procedures, control plates (two malt extract agar (MEA) and two lysogeny broth agar (LBA)) were employed in the laminar flow hood. Each plate was placed at one corner and exposed to the environment in the hood. They were incubated along with the culture plates and observed regularly for any growth.

### 3.5. PCR Amplification

DNA from the fungal and bacterial isolates obtained by culturing was subjected to polymerase chain reaction (PCR) amplification. DNA was extracted using a CTAB (cetyltrimethylammonium bromide) extraction method [53,54,55]. ITS4 and ITS5 primers were used to amplify the ribosomal DNA (rDNA) internal transcribed spacers (ITS1 and ITS2) and the 5.8S gene in fungi [56]. For bacterial isolates, primers 16S-2 and 23S-7 were used to amplify the rDNA intergenic spacer region (ITS1), along with portions of the small subunit and large subunit genes [57]. A GeneAmp PCR Reagent kit (Applied Biosystems, Carlsbad, CA, USA) was used for amplification. The composition of each 50 μL reaction was as follows: five microliters of cell suspension, 50 pmol of each primer, 10 pmol of each dNTP, 2U *Taq* DNA polymerase, 50 mM KCl and 1.5 mM MgCl_2_. The program cycle used for amplification was: 95 °C for 8 min, 40 cycles of 94 °C for 1 min, 54 °C for 1 min 30 s and 72 °C for 2 min, followed by 72 °C for 8 min, and finally cooled to 4 °C. PCR reactions were subjected to electrophoresis on 1% agarose gels with TBE (90 mM Tris-Borate, 2 mM EDTA, pH 8.0) and 0.5 μg/mL ethidium bromide was used to view the amplification using UV light. For PCR amplification, sterilized water was used as a negative control. *Rhodotorula mucilaginosa* was used as the positive control for PCR with fungal primers and *Bacillus subtilis* as positive control for bacterial PCR.

### 3.6. Cloning and Sequencing

A TOPO TA cloning kit (Invitrogen, Grand Island, NY, USA) was used for cloning the amplified products. The amplified DNA from the fungal and bacterial isolates was ligated into the PCR 4-TOPO vector. The ligation reaction included: two and a half microliters of PCR product, 1.0 μL of salt solution (200 mM NaCl, 10 mM MgCl_2_) and 1.5 μL of vector (10 ng μL^−^^1^). The ligation reaction was carried out following the manufacturer’s directions. One Shot^®^ TOP10 Competent *E. coli* cells were transformed with the ligation reaction. The plasmid DNA was isolated from transformed cells using the Cyclo-Prep Plasmid DNA isolation kit (Amresco, Solon, OH, USA). The plasmids then were tested for the presence of inserts using *Eco*RI digestion. The products were subjected to electrophoresis on 1% agarose gels with TBE and 0.5 μg/mL ethidium bromide to analyze the digestion products. Plasmids containing the inserts then were sent to Gene Gateway LLC (Hayward, CA, USA) for sequencing.

### 3.7. Direct Sequencing of Cultures

After subculturing, DNA was extracted from the growing cultures of meltwater. The extracted DNA was amplified using a GeneAmp (with Ampli *Taq*) PCR Reagent Kit (Applied Biosystems, Carlsbad, CA, USA). A final concentration of each reaction was 5 pmol of dATP, dCTP, dGTP, dTTP, 1× PCR Buffer [10 mM Tris-HCl, pH 8.3, 50 mM KCl, 1.5 mM MgCl_2_, 0.001% (w/v) gelatin], 1 unit Ampli *Taq* DNA polymerase and fungal rRNA ITS and bacterial rRNA SSU primers, in 25 µL total volume. Each reaction had 25 pmol of a forward and 25 pmol of a reverse primer. The reaction was carried in a thermocycler and the program was: 94 °C for 4 min; then 40 cycles of 94 °C for 1 min, 55 °C for 3 min, 72 °C for 3 min; followed by an incubation for 10 min at 72 °C. After amplification the samples were quantified on 1% agarose gels in TBE (as above). Samples were cleaned using a Exo-SAP IT kit (Affymetrix, Inc., USB Products, Cleveland, OH, USA). Exo-SAP IT is composed of Exonuclease I and Shrimp Alkaline Phosphatase. It is a special buffer that removes any remaining primer and dNTPs from the PCR reaction. Samples were sent to GENEWIZ (South Plainfield, NJ, USA) for Sanger sequencing. They were examined and aligned using Clustal X or MAFFT [58,59], and then were subjected to phylogenetic analysis using maximum parsimony with PAUP [30].

### 3.8. Metagenomic/Metatranscriptomic Analyses

#### 3.8.1. Ultracentrifugation

Two samples were selected for metagenomic/metatranscriptomic analysis, GISP2D 1,601 m and 3,014 m. The former sample corresponds to 10,500 ybp [22,24,26], a time of moderate and rising atmospheric CO_2_ and rising temperatures, while the latter corresponds to 157,000 ybp, a time of low atmospheric CO_2_ and low temperatures [50]. Starting from Shell 2 inward, the meltwater was subjected to ultracentrifugation at 32,500 rpm in a Beckman type 60 Ti rotor (100,000 rcf) at 4 °C for 15–17 h to concentrate cells, viruses and nucleic acids. The supernatants were placed into sterile BD Falcon 50 mL conical tubes and stored at −20 °C. Each pellet was resuspended in 50 µL of 0.1× TE (1 mM Tris HCl, pH 8.0; 0.1 mM EDTA, pH 8.0) and then the suspensions from each tube (for a given meltwater sample) were pooled (~500 µL total) and stored at −20 °C. Two additional samples were used as controls. The first control was autoclaved Nanopure water (18.2 MΩ, <1 ppb TOC) that was subjected to ultracentrifugation, as above. Nanopure water (18.2 MΩ, <1 ppb TOC) was the second control.

#### 3.8.2. Isolation of Nucleic Acids

Each concentrated meltwater sample (as well as the two controls) was divided into two 200 µL fractions. One fraction was used for RNA extraction using TRIzol LS reagent (Life Technologies, Grand Island, NY, USA). To carry out homogenization, three volumes of TRIzol LS reagent were added, mixed and incubated to allow complete dissociation of proteins. Next, an equal volume of chloroform was added, followed by vigorous mixing. After incubation at room temperature, the samples were centrifuged in a microfuge at 16,100 rcf, which resulted in three layers. The top aqueous layer, containing the RNA, was removed and the RNA was precipitated by adding 400 µL of isopropanol. This was placed at −20 °C for 2 h, followed by centrifugation in a microfuge to pellet the RNA. This pellet was washed with 800 µL 80% ethanol, air-dried and stored at −80 °C until needed. Directly before use, the RNA pellet was resuspended in 20 µL of sterile RNAse free water. The second 200 µL fraction of pooled ultracentrifuged meltwater was used for DNA extraction using a CTAB method, as described above.

#### 3.8.3. Preparation of Samples for Sequencing

Complementary DNAs (cDNAs) were synthesized from the extracted RNAs. The procedure was performed using SuperScript Choice cDNA kit (Invitrogen, Grand Island, NY, USA), according to the manufacturer’s instructions, using 10 µL of the extracted RNA and 80 pmol of random hexamer primers. The cDNA was then mixed with 10 µL of extracted DNA (less than 1 ng/µL) from the same meltwater sample, and *Eco*RI (*Not*I) adapters (AATTCGCGGCCGCGTCGAC, dsDNA) were added using T4 DNA ligase. The final concentration of components in each reaction for *Eco*RI adapter addition was: 66 mM Tris-HCl (pH 7.6), 10 mM MgCl_2_, 1 mM ATP, 14 mM DTT, 100 pmols *Eco*RI (*Not*I) adapters and 0.5 units of T4 DNA ligase, in 50 µL total volume. The reaction was incubated at 15 °C for 20 h. Then, the reaction was heated to 70 °C for 10 min to inactive the ligase. [Note: The cDNA were mixed in order to maximize the biomass of nucleic acids, necessary for successful pyrosequencing. Thus, the cDNA comprises a metatranscriptomic fraction and the DNA comprises the metagenomic fraction of each sample].

The products were size fractionated by column chromatography. Each 2 mL plastic column contained 1 mL of Sephacryl^®^ S-500 HR resin. TEN buffer (10 mM Tris-HCl [pH 7.5], 0.1 mM EDTA, 25 mM NaCl; autoclaved) was utilized in washing the columns and eluting the samples through the columns. Fractions of approximately 40 µL were collected by chromatography. After measurement of the volume of each fraction, fractions 6–18 were precipitated to concentrate the DNA. Concentration consisted of adding 0.5 volumes (of the fraction size) of 1 M NaCl, and two volumes of −20 °C absolute ethanol. After gentle mixing, each was left to precipitate at −20 °C overnight. The fractions were centrifuged in a microfuge for 20 min at room temperature and decanted. Then, the pellets were washed with 0.5 mL of −20 °C 80% ethanol and centrifuged for 5 min. Finally, each of the DNA pellets was dried under vacuum and rehydrated in 20 µL of 0.1× TE buffer.

After resuspension, fractions 6–18 were subjected to PCR amplification using *Eco*RI (*Not*I) adapter primers (AATTCGCGGCCGCGCTCGAC). The samples were amplified using a GeneAmp PCR Reagent Kit (as described above). The thermal cycling program was 94 °C for 4 min; then 40 cycles of 94 °C for 1 min, 55 °C for 2 min, 72 °C for 2 min; followed by an incubation for 10 min at 72 °C. Each fraction was subjected to gel electrophoresis on 1% agarose gels in TBE (as above) to confirm amplification, and to determine the size distributions. 

454-specific primers were used to amplify the fragments, which also added the specific 454 sequences to the ends of the amplified products. All of the primers included the A-tag or the B-tag sequence (CGTATCGCCTCCCTCGCGCCA, CTATGCGCCTTGCCAGCCCGC, respectively), as well as the short four-base tag sequence (TCAG), unique multiplex identifiers (MID sequences (MID4: AGCACTGTAG, MID5: ATCAGACACG, MID6: ATATCGCGAG, MID10: TCTCTATGCG)), and *Eco*RI (*Not*I) adapter sequences (AATTCGCGGCCGCGTCGAC). The samples were amplified using a GeneAmp PCR Reagent Kit (as described above). All PCR products were cleaned with a PCR purification kit (Qiagen, Valencia, CA, USA). Once the sizes to be used for sequencing were confirmed by gel electrophoresis, the fractions containing distributions from approximately 200 bp to 1.5 kb were pooled. After concentration by precipitation and rehydration, the samples were quantified on 1% agarose gels in TBE (as above), using serial dilutions of plasmid (pGEM-3Z, from Promega, Madison, WI, USA) for quantitation.

#### 3.8.4. Sequencing

DNA concentrations were estimated to be ~150 ng/µL for GISP 2D 3,014 m (sample 1); ~200 ng/µL for GISP 2D 1,601 m (sample 2); ~150 ng/µL for the ultracentrifuge sterile Nanopure water (sample 3); and finally the ~250 ng/µL for the unconcentrated Nanopure water. These were mixed in equimolar amounts in the proportion of 4:3:4:2, respectively, based on their estimated concentrations. Eight µL of Sample 1 (GISP 2D 3,014 m), 6 µL of Sample 2 (GISP 2D 1,601 m), 8 µL of Sample 3 (control ultracentrifuged water), and 4 µL of Sample 4 (control water) were mixed to produce a composite sample for sequencing. To this 26-µL mixture, 28 µL of RNase free water was added. A total of 54 µL (4.6 mg) of the amplified DNA at an approximate concentration of 85 ng/mL was sent to the University of Pennsylvania Medical School Sequencing Facility for Roche/454 GS FLX service.

#### 3.8.5. Analysis of the Metagenomic/Metatranscriptomic Sequences

The sequences were assembled using MIRA 3.0.5 [60] and then were separated into four files, based on their multiplex identifier (MID) sequences (corresponding to the four ice core and control samples). Then, the 454 primers were clipped off (in silico) and sequence assembly was performed. The assembled sequences were used in Megablast searches for determination of sequence, taxon and gene similarities. The Megablast results (with the cut off parameter 1e-10) were retrieved for further analysis. Database sequences were reorganized based on gene regions. Multiple sequence alignments for ribosomal RNA small subunit (rRNA SSU) and large subunit (rRNA LSU) genes, separately, were performed with MAFFT (Multiple Sequence Alignment based on Fast Fourier Transform, Kyoto University, Kyoto, Japan) [58,59,61] global and local alignment tools. Alignment files were converted into NEXUS format with SeaView (Université de Lyon, Villeurbanne, France) [62,63]. Sequences from bacterial isolates CULT A-1 and CULT B-1 were included in the phylogenetic analyses. PAUP (Phylogenetic Analysis Using Parsimony, [30]) maximum parsimony phylogenetic reconstructions were performed on the aligned SSU and LSU rRNA gene sequences. Bootstrap analyses (1,000 replications) were used to examine the support for the branches. Results from the phylogenetic analyses of the sequences from uncultured samples allowed a reevaluation of the microbial compositions of the two ice samples.

### 3.9. Phylogenetic Analysis for Fungal and Bacterial Sequences

The sequences obtained from cultures were subjected to BLAST searches and phylogenetic analysis in the same manner as the metagenomic sequences. The rRNA gene sequences obtained from both the bacterial and fungal isolates were used in BLAST searches of the GenBank NCBI-database to identify sequences of related taxa. Sequence alignments were created using ClustalX 2.0 for bacteria and fungi using the isolate sequences and related NCBI sequences. Alignment files were used to generate maximum parsimony phylogenetic trees using the program PAUP [30]. The phylogenetic trees were created using the heuristic search option, and gaps were treated as a fifth base. Bootstrap support using 1,000 replications also was determined using the same criteria.

## 4. Conclusions

A variety of microbes and their nucleic acids were recovered from Greenland and Antarctica ice core sections. In general, the viability of microbes decreased with increasing ice core age (Figure 4). While similar species were found in Arctic and Antarctic ice of the same age, the species differed, and in all cases the species composition within each ice core section was unique (Table 1, Figure 6, Figure 7, Figure 8). Among the bacteria, species of *Bacillus* were common, while among the fungi, species of *Rhodotorula* were the most common. Cultivation and metagenomic/metatranscriptomic studies indicated that viability was dependent on cold temperatures at the time of deposition (Figure 5). Low concentrations of atmospheric dust and CO_2_ also were related to increased viability.
